# Prediction of CTL epitope, *in silico* modeling and functional analysis of cytolethal distending toxin (CDT) protein of *Campylobacter jejuni*

**DOI:** 10.1186/1756-0500-7-92

**Published:** 2014-02-19

**Authors:** Arun G Ingale, Susumu Goto

**Affiliations:** 1 Department of Biotechnology, School of Life Sciences, North Maharashtra University, Jalgaon 425001, India; 2 Bioinformatics Centre, Institute of Chemical Research, Kyoto University, Kyoto, Japan

**Keywords:** Cytolethal distending toxin (CDT), CTL epitope prediction, *Campylobacter jejuni*, Homology modeling

## Abstract

**Background:**

*Campylobacter jejuni* is a potent bacterial pathogen culpable for diarrheal disease called campylobacteriosis. It is realized as a major health issue attributable to unavailability of appropriate vaccines and clinical treatment options. As other pathogens, *C. jejuni* entails host cellular components of an infected individual to disseminate this disease. These host–pathogen interfaces during *C. jejuni* infection are complex, vibrant and involved in the nicking of host cell environment, enzymes and pathways. Existing therapies are trusted only on a much smaller number of drugs, most of them are insufficient because of their severe host toxicity or drug-resistance phenomena. To find out remedial alternatives, the identification of new biotargets is highly anticipated. Understanding the molecules involved in pathogenesis has the potential to yield new and exciting strategies for therapeutic intervention. In this direction, advances in bioinformatics have opened up new possibilities for the rapid measurement of global changes during infection and this could be exploited to understand the molecular interactions involved in campylobacteriosis.

**Methods:**

In this study, homology modeling, epitope prediction and identification of ligand binding sites has been explored. Further attempt to generate strapping 3D model of cytolethal distending toxin protein from *C. jejuni* have been described for the first time.

**Results:**

CDT protein isolated from *C. jejuni* was analyzed using various bioinformatics and immuno-informatics tools including sequence and structure tools. A total of fifty five antigenic determinants were predicted and prediction results of CTL epitopes revealed that five MHC ligand are found in CDT. The three potential pocket binding site are found in the sequence that can be useful for drug designing.

**Conclusions:**

This model, we hope, will be of help in designing and predicting novel CDT inhibitors and vaccine candidates.

## Background

*Campylobacter jejuni* is a prominent bacterial cause of enteric campylobacteriosis in the entire world [1]. *Campylobacter* is extensively distributed in poultry; nevertheless, cattle, pigs, sheep, and pet animals may also be a source of these microorganisms. This infection may be due to either eating of semi cooked meat or cross-contamination of ready-to-eat food at the time of preparation or storage. *C. jejuni*-linked enterocolitis is characteristically coupled with a local acute inflammatory response that involves intestinal tissue damage [2]. The genome of *C. jejuni* has been sequenced, yet only a few prospective virulence factors produced by *C. jejuni* are well considered [3].

Cytolethal distending toxins (CDT) are a class of heterotrimeric toxins produced by *C. jejuni* and also by closely related spp., such as *C. fetus*, *C. coli *[4,5], *Shigella *[6] and *Escherichia coli *[7]. This toxin is rearward transported across the golgi complex and the endoplasmic reticulum, and afterward translocated into the nuclear compartment, where it applies the toxic activity [8]. The CDT comprises of three protein subunits namely CdtA, CdtB, and CdtC causes progressive cellular distention with ultimate cell death and have been proposed as virulence factors in the pathogenesis of *C. jejuni *[9]. These results suggest that the CDTs are involved invasion, survival and internalization into the host cell [10-13]. Although CDT from *C. jejuni* has been studied and characterized in laboratory [14,15], but research on immune responses and pathogenesis of *C. jejuni* remains unexploited.

The progress in computational methods competent of predicting immune epitopes for B lymphocytes and T lymphocytes will facilitate the viewing of pathogens for immunogenic antigens. The epitope based vaccines encourage an immune response by presenting immunogenic peptides unite to major histocompatibility complex to TCR [16]. Considering the unavailability of 3D structure of CDT, it is challenging to select proper target that would lead to predict epitope and ligand binding sites in protein. Hence, this study aims to investigate the CDT of *C. jejuni* with special focus on the structural and functional aspects through bioinformatics approach. This study has important implications on the selection of CTL epitope, a critical step in the development of vaccines.

## Methods

### Sequence acquisition and analysis

We have received the sequence of CDT of *C. jejuni* from the NCBI database by inserting query as “CDT *C. jejuni*”. The sequence was saved in FASTA format and used for further analysis. The primary structure analysis was done by using expasy ProtParam (http://www.expasy.org). The secondary structure of the protein was computed using different servers like Jpred3, GOR-IV and SOPMA [17] to check the presence of alpha helix and beta plated sheets in the structure. To determine the possible function of *C. jejuni*, the sequence was subjected to comparative protein structure modeling in the different servers.

### 3D-Model building and validation

Cytolethal distending toxin sequence of *C. jejuni* (CDTCJ) [EDZ32284.1] was used to develop 3D structure through homology modeling because crystal or NMR structure of the CTD protein was not available in the Protein Data Bank (PDB). The 3D structure of the CDT protein was done using a restrained-based approach in Modeller. The 3D model was generated using the ModWeb server that generates 3D models along with their confidence score (C-Score). The template selection for the homology modeling of the CDT protein was performed by submitting amino acid sequence of the target protein to ModWeb server [18]. The crystal structure of CDT from *Haemophillus ducreyi* (PDB ID:1SR4) was used as a template. After generating the 3D model, structure analysis and stereochemical analysis were performed using different evaluation and validation tools. The final model was validated by using SAVES online tool (http://nihserver.mbi.ucla.edu/SAVES/). The Ramachandran plot was obtained using PROCHECK [19] and RAMPAGE [20] which helped in evaluating backbone conformation. Ramachandran plot was also used to check non-GLY residues at the disallowed regions. The verify 3D and PROSA web tool [21] was used to determine Z-scores. The ERRAT was used to predict overall quality for model and quality of the model was assured using Z- scores.

### Epitope prediction of protein antigens

SEPPA (Spatial Epitope Prediction of Protein Antigens) server at the Life Science and Technology School, Tongji University, Shanghai China, (http://lifecenter.sgst.cn/seppa/) was used to predict conformational B-cell epitope.

The 3D protein structure predicted by Modeller was used as an input, each residue in the query protein will be given a score according to its neighborhood residues information. Higher score corresponds to higher probability of the residue to be involved in an epitope [22]. The default values of THRESHOLD was set at 1.80, this help to specify the epitope residues [23]. Transmembrane topology of the CDTCJ protein was checked using TMHMM online tool [24] and antigenicity of protein was checked using SVMTriP online antigen prediction server [25]. The several algorithms are available that can predict the location and binding specificity of CTL epitopes in the protein sequences. In this study, the cytotoxic T-lymphocyte epitope prediction was done using NetCTL-1.2 server [26].

### Sub cellular localization prediction

The sub cellular localization of CDT was predicted using CELLO, an approach based on multi-class SVM classification system [27]. CELLO uses four types of sequence coding schemes: the amino acid composition, the di-peptide composition, the partitioned amino acid composition and the sequence composition based on the physico-chemical properties of amino acids. TargetP1.1 server was also used to predict cleavage site prediction of CDT [28].

### Protein interaction network mapping

Protein-protein interactions were achieved from the STRING database [29] comprising known and predicted physical and functional protein-protein interactions. STRING in protein mode was used, and only interactions with high confidence levels (>0.7) were kept. STRING quantitatively integrates interaction data from these sources for many organisms, and transfers information among these organisms where applicable. Network visualization was done with the Cytoscape software [30].

### Ligand binding sites prediction

We used MetaPocket 2.0 server (http://metapocket.eml.org) to identify ligand-binding sites on the protein surface. The MetaPocket is a consensus method [31] developed at Technical University of Dresden and Zhejiang University jointly, in which the predicted binding sites from eight methods *i.e.*, PASS11 (PAS), LigsiteCS (LCS), Q_SiteFinder (QSF), GHECOM (GHE), POCASA (PCS), Fpocket (FPK), SURFNET (SFN), ConCavity (CON) are combined to improve the prediction success rate.

### Structure comparison

The structure comparison was executed by using DaliLite server [32].

## Results and discussion

**Figure 1:**
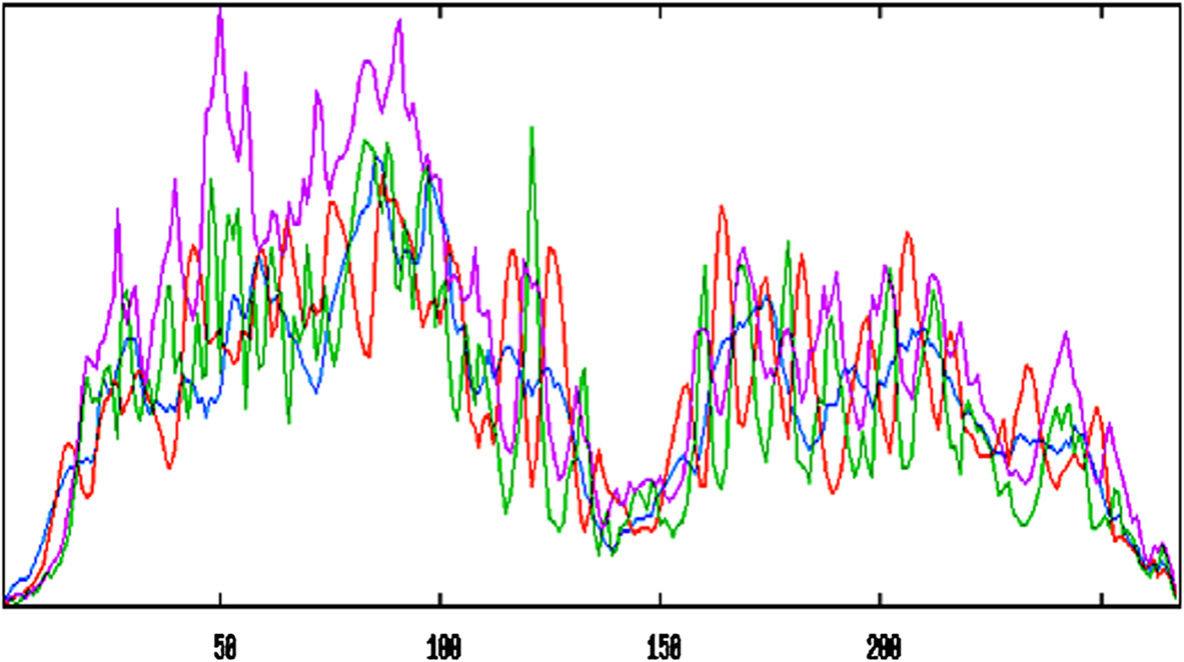
**Secondary structure of CDT of *C. jejuni.***

**Figure 2:**
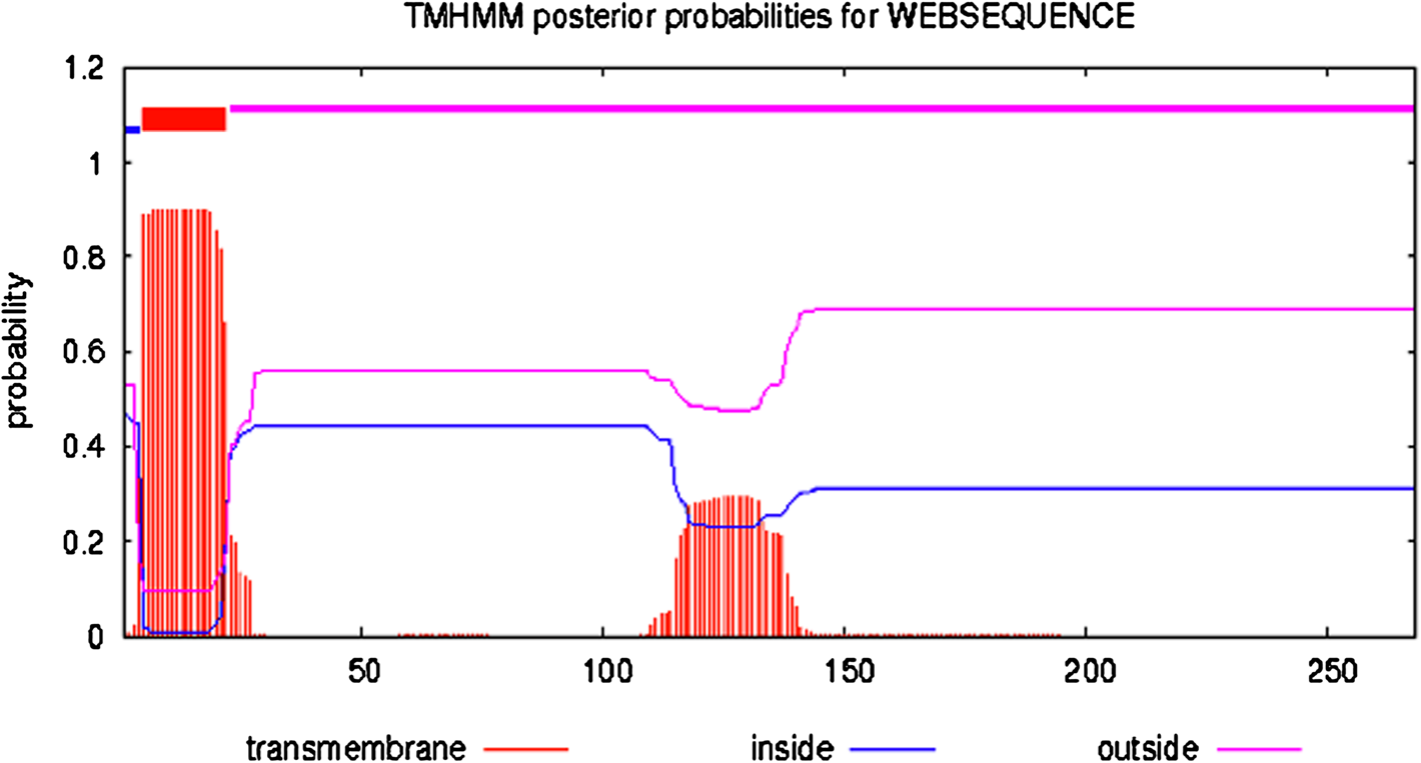
**Transmembrane topology of CDTCJ of *C. jejuni.***

The current study was originated to perform structure based sequence analysis of the CDT protein isolated from *C. jejuni*. The protein sequence was obtained from the NCBI protein database using accession number gi|205345645|gb|EDZ32284.1| cytolethal distending toxin [*Campylobacter jejuni*]. Primary structure analysis revealed that the CDT protein (268 aa) had a molecular weight of 29.94 kD and theoretical isoelectric point (PI) 6.81. An isoelectric point indicates a negatively charged protein. The instability index (II) was 18.60, thereby categorizes the protein as a stable. The aliphatic index appeared as 84.10 and the N-terminus of the sequence showed the presence of M (Met). The negative grand average of hydropathicity (GRAVY) of -0.061 denoted that the protein was hydrophillic. The amino acids, Asn (N), Phe (F), Ala (A), and Leu (L), were found in high praportion in the protein. The secondary structure disclosed the presence of 8.21% α-helices, 4.85% β-turns, 25.37% extended strand and 61.57% coils (Figure 1). Transmembrane topology of the CDTCJ protein was checked using TMHMM online tool. The TMHMM server showed that residues 23-268 presented outside region, residues 5-22 were within the transmembrane and residues 1-4 were inside the region of the protein. Hydropathy analysis of CDTCJ protein of *C. jejuni* by the TOPCONS [33], Signal P-4.0 [34] and TMHMM programs suggested the presence of only one TM helix. We therefore localized the N terminus of CDTCJ in the cytoplasm. A consensus predicted topology is presented in Figure 2.

The sub cellular localization of CDT was predicted using CELLO, an approach based on a two-level support vector machine (SVM) system. This server predicts sub cellular localization of protein for Gram negative bacteria by supporting vector machines based on n-peptide compositions. The CELLO output gave significant reliability for outer membrane (0.198), periplasmic (1.76) extracellular (0.803) and cytoplasmic (2.493), it indicates that the protein is cytoplasmic.

### Model function and validation

**Figure 3:**
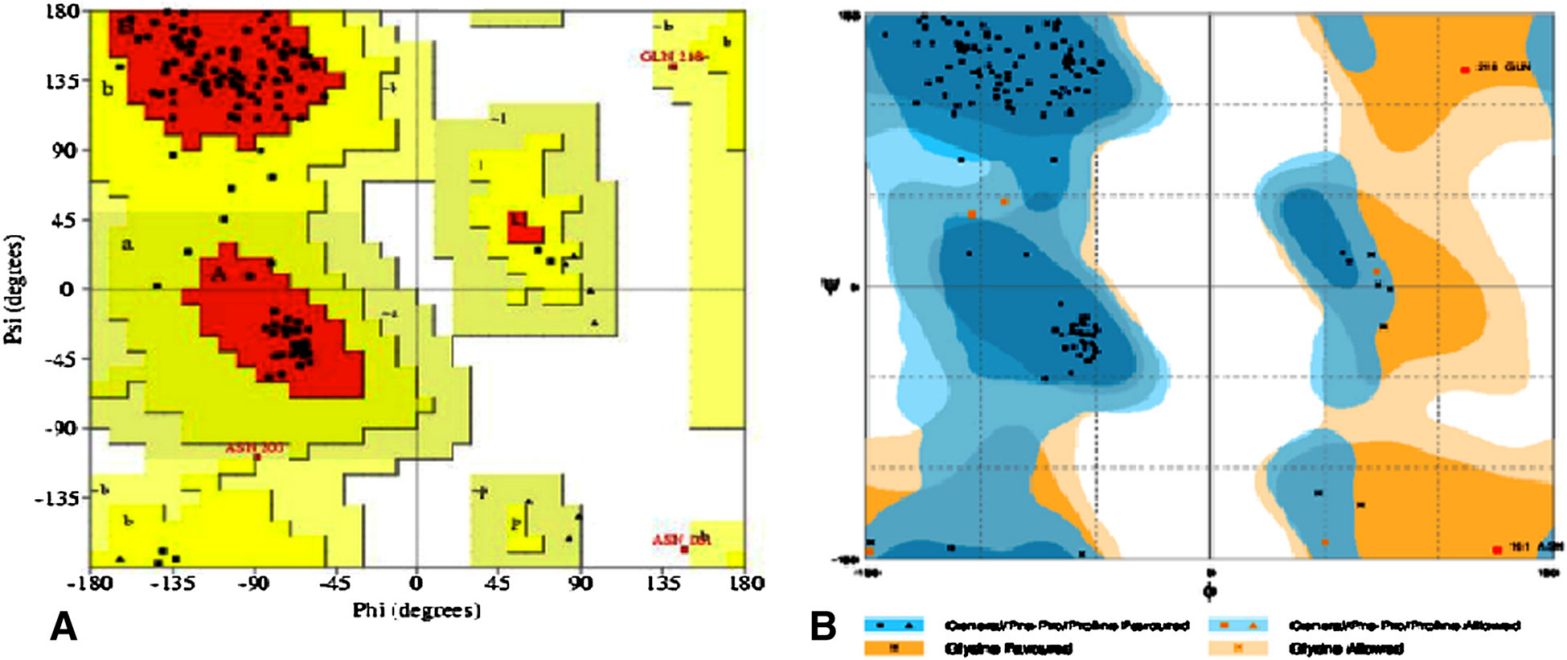
**The Ramchandran plot of structure of CDTCJ.** Showing residue predicted by PROCHECK **(A)** and RAMPAGE **(B)**. Results of CDTCJ protein showing residues in favored, allowed outlier regions.

**Figure 4:**
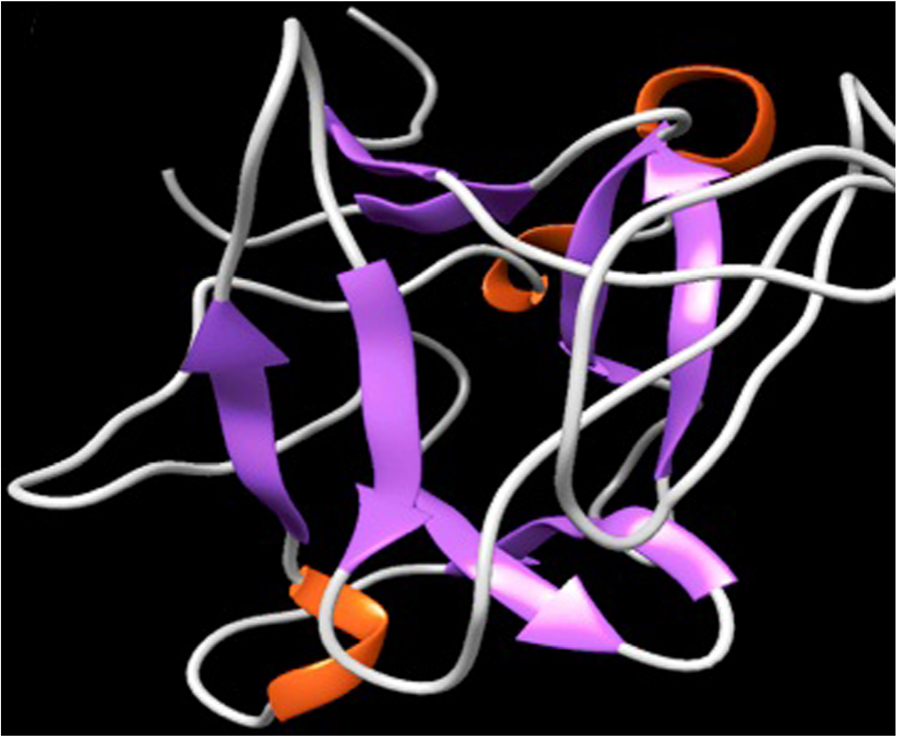
**Homology model of CDTCJ.** 3D structure of CDTCJ protein visualized by UCSF CHIMERA visualizing tool. The cartoon representation of 3D modeled structure of CDTCJ using PDB ID: 1SR4 shows helix (orange), sheets (purple) and loops (sky blue).

To determine the possible function of CDT, the sequence was subjected to comparative protein structure modeling using the target protein sequence as query for different servers described in Methods. The modeling of CDTCJ was performed using a restrained-based approach implemented in MODWEB [35] and significant hits were obtained. A set of three models for CDT protein was constructed. The 3D structure of a CDTCJ protein was developed from the X-ray structure of *Haemophilus ducreyi* (PDB ID: 1SR4 Chain A, at 2.0 Å resolution) as a template for homology modeling. The alignment coverage region for target residue (113–258) showed the 37% sequence identity with template 1SR4 residue 75–219. The resulting 3D models of CDTCJ were sorted according to the scores calculated from discrete optimized protein energy (DOPE) scoring function. The final model that shared the lowest root mean square deviation (RMSD), relative to the trace (Ca atoms) of the crystal structure was selected for further studies. The validation of the model was performed by accessing the quality of backbone conformation by PROCHECK for reliability. The perceived Ramchandran plot (Psi-Phi) pairs had 86.5% of residues in most favored regions, 11.1% core residues in additional allowed regions, 1.6% residues in generously allowed regions and 0.8% residues in disallowed regions (Figure 3). These values indicated a good quality model. Whereas the crystal structure of *Haemophilus ducreyi* PDB ID 1SR4 shows 89% residue in most favor region [36]. To characterize the model, structural motif and mechanistically important loops were assigned to build the final 3D model of CDTCJ. The 3D model of CDTCJ using the template 1SR4, consist of two domains that encompasses 8β-sheets and 3α-Helices (Figure 4). Verify3D and ERRAT were also used to further assess the quality of the CDTCJ model. Verify3D analyzes the compatibility of the model against its own amino acid sequence and results revealed that 59.86% of the residue had an average 3d-1D score 0.2. Verify3D and ProSA gave good scores for overall model quality. However, the ERRAT validation of CDTCJ model indicated regions where the calculated errors were higher than expected that decreases the overall quality score to 46.7%.

### Structure comparison analysis

**Figure 5:**
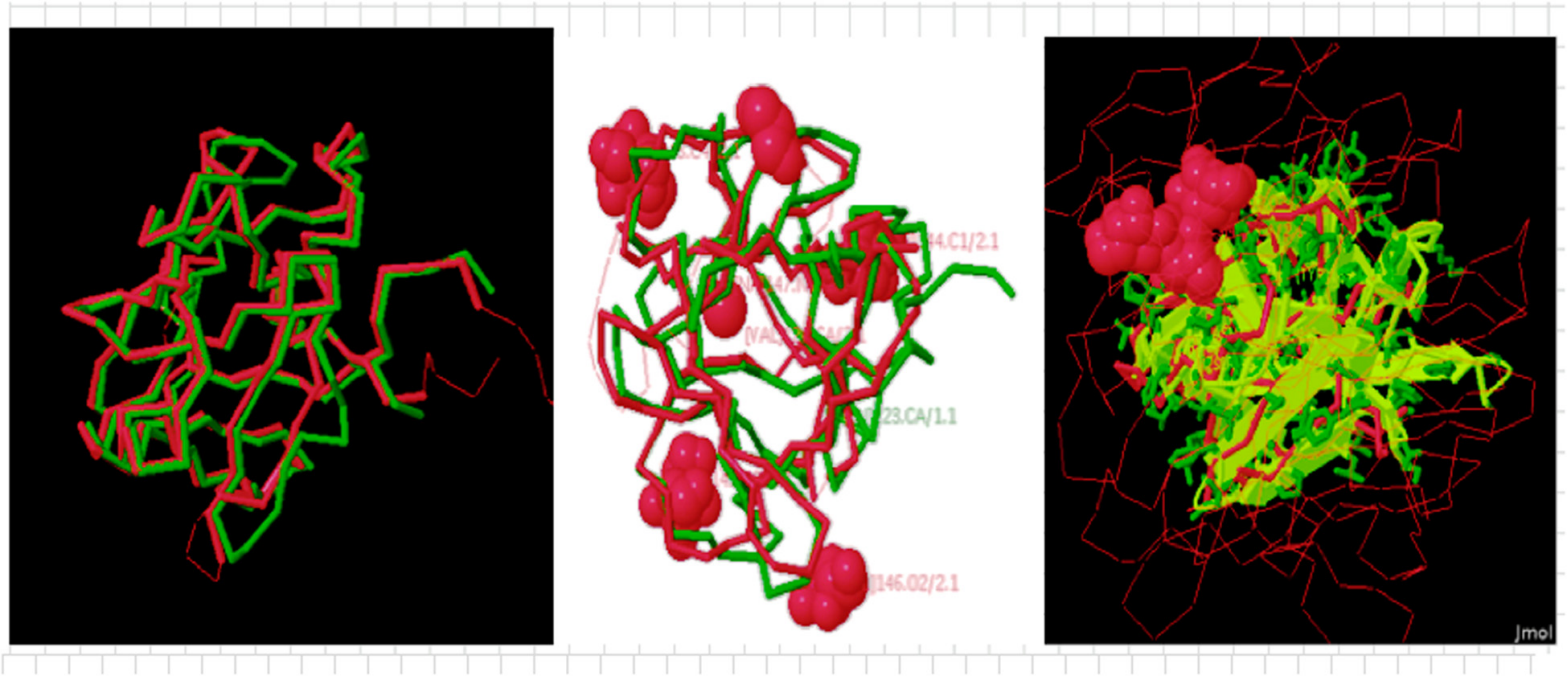
The superimposition of 3D model of CDTCJ using Dalilite v.3.3.

Comparative analysis of CDTCJ structure was performed using DaliLite v.3.3. server. This server is a network service for comparing protein structure in 3D and computes optimal and suboptimal structural alignments between two protein structures. It helps in understanding the fundamental role of proteins and their functions. The structural similarity relationships among protein structures allow users to infer the functions of newly discovered proteins [37]. The final refined model of CDTCJ was superimposed with template by using DaliLite. The superimposition of model to the template is shown in Figure 5. The result provided by DaliLite servers show the 851 alignments with compatible Z-score. The highest Z-score for structure from PDB ID: 2F2F, 1SR4 was 28.3, 27.5 and percent identity 38, 37 respectively. It is interesting to note that first two high Z-score proteins are 2F2F and 1SR4, were also used for the development of model 3D structure.

### Epitope prediction of protein antigens

**Figure 6:**
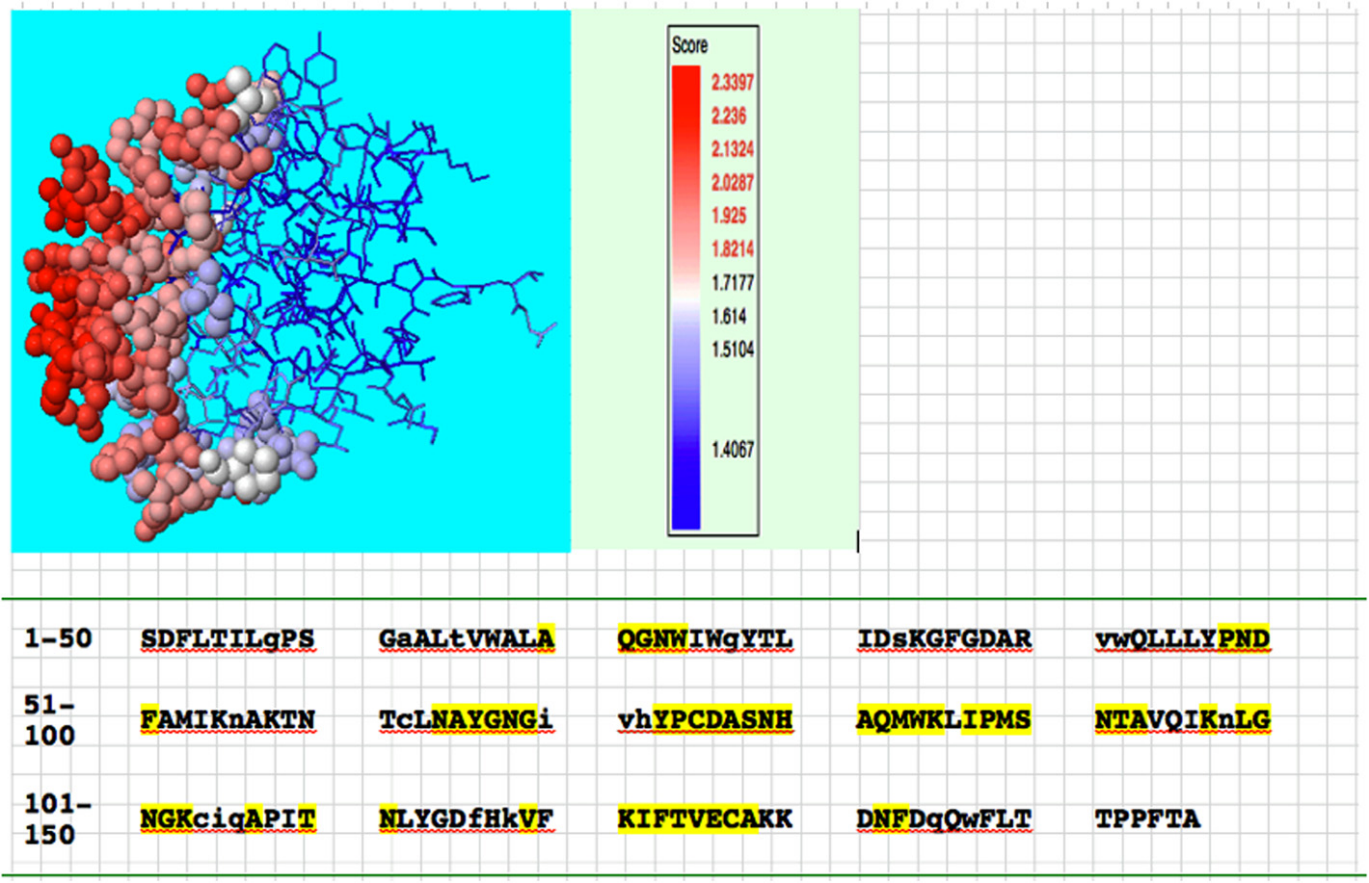
**Antigenic epitope sites predicted by SEPPA server.** The red sphere shows highest antigenicity residue and blue ones are less antigenic.

Potentially immunogenic regions of CDTCJ were predicted by using the SEPPA server. This server analyses 3D structures and aims at the division of antigens surface in epitopic and non epitopic patches on the basis of different propensity scores and solvent accessibility; they all rely on training datasets comprising resolved antibody/antigen complexes [38]. A total of 55 epitopes were predicted from 146 aa using default threshold value of 1.80. The predicted epitopes visualized with JMOL in different renderings are shown in Figure 6. In this structure, tints from blue to red represent a rising antigenicity. Highlighted epitope residues were predicted and shown in red solid spheres. The prediction results are also displayed in a table and each, residue is listed sequentially. The predicted epitope residues are highlighted in yellow and the core residues are shown in lowercase. Antigenic epitopes that are preferentially recognized by antibodies that can help in the design of vaccine components and immuno-diagnostic reagents [39].

### Cytotoxic T-Lymphocytes (CTL) epitopes

Epitope predictors are routinely tested on large sets of epitopes derived from various pathogens. Schellens *et al.*[40] identified eighteen new CTL epitopes out of a set of twenty two predicted CTL epitopes in HIV-1 using NetCTL. We screened all possible peptide fragments of 9aa within a particular protein, and eliminated those fragments that cannot be correctly processed by either the proteasome, TAP or the MHC class I molecules. Prediction results of CTL epitopes revealed that five MHC ligands were found in CDT sequence having high e-value score are positioned at _10_CCFMTFFLY_18_, _39_DTDPLKLGL_47_, _132_AQGNWIWGY_140_, _170_KTNTCLNAY_178_ and _217_IQAPITNLY_225_. These are the immunodominant epitopes restricted by MHC class I located arbitrarily in the protein sequence. This data indicate that CTL epitopes in CDT are randomly distributed, and this distribution is similar to those of CTL epitopes in proteins from other proteomes.

### Protein interaction network mapping

**Figure 7:**
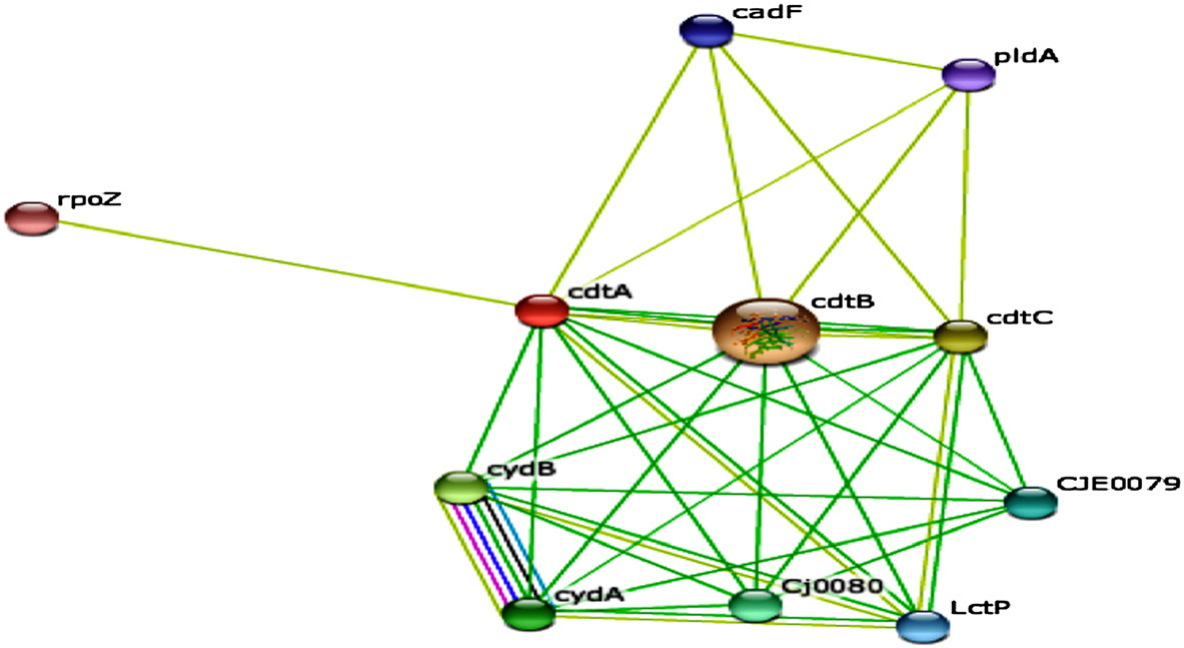
**Interaction network of CDTCJ produced by STRING database.** In this network, CDTCJ protein showed the highest interaction score 0.920 with CDTCJ-B protein.

To compute protein-interaction properties of the CDT, we used the search tool for the retrieval of interacting genes and proteins (STRING) database of physical and functional interactions [41]. The prediction of CDTCJ interactions using protein structural similarities permit to construct various candidates interactions with possibly significant functional relevance. For this purpose, relation among the ten identified proteins was examined. The interaction network for genetically interacting proteins possibly related in function with *C. jejuni* is shown in Figure 7, and the detail information is presented in Table 1. Green lines indicate co-localization in genomes (likely operon structures), and blue lines indicate statistically significant co-occurrence across multiple genomes. A graph of the CDTCJ network shows the identified CDTCJ-interacting proteins and phylogenomic profiling of CDT-related functions.

**Table 1:** **List of predicted interactive proteins with CDTCJ of *C. jejuni***

**Sr. No**	**ID**	**Protein name**	**Amino acid residue**	**Score**
1	cdtB	Cytolethal distending toxin, subunit B	265	0.920
2	cdtC	Cytolethal distending toxin, subunit C	189	0.897
3	cydB	Cytochrome d ubiquinol oxidase, subunit II	374	0.651
4	cydA	Cytochrome d ubiquinol oxidase, subunit I	520	0.651
5	Cj0080	Hypothetical protein	89	0.651
6	Cje0079	Hypothetical protein	34	0.628
7	LctP	L lactate permease	565	0.614
8	cadF	Fibronectin binding protein	319	0.569
9	pldA	Phospholipase A	329	0.517
10	rpoZ	DNA directed RNA polymerase, Subunit omega	74	0.514

### Ligand binding sites

**Figure 8:**
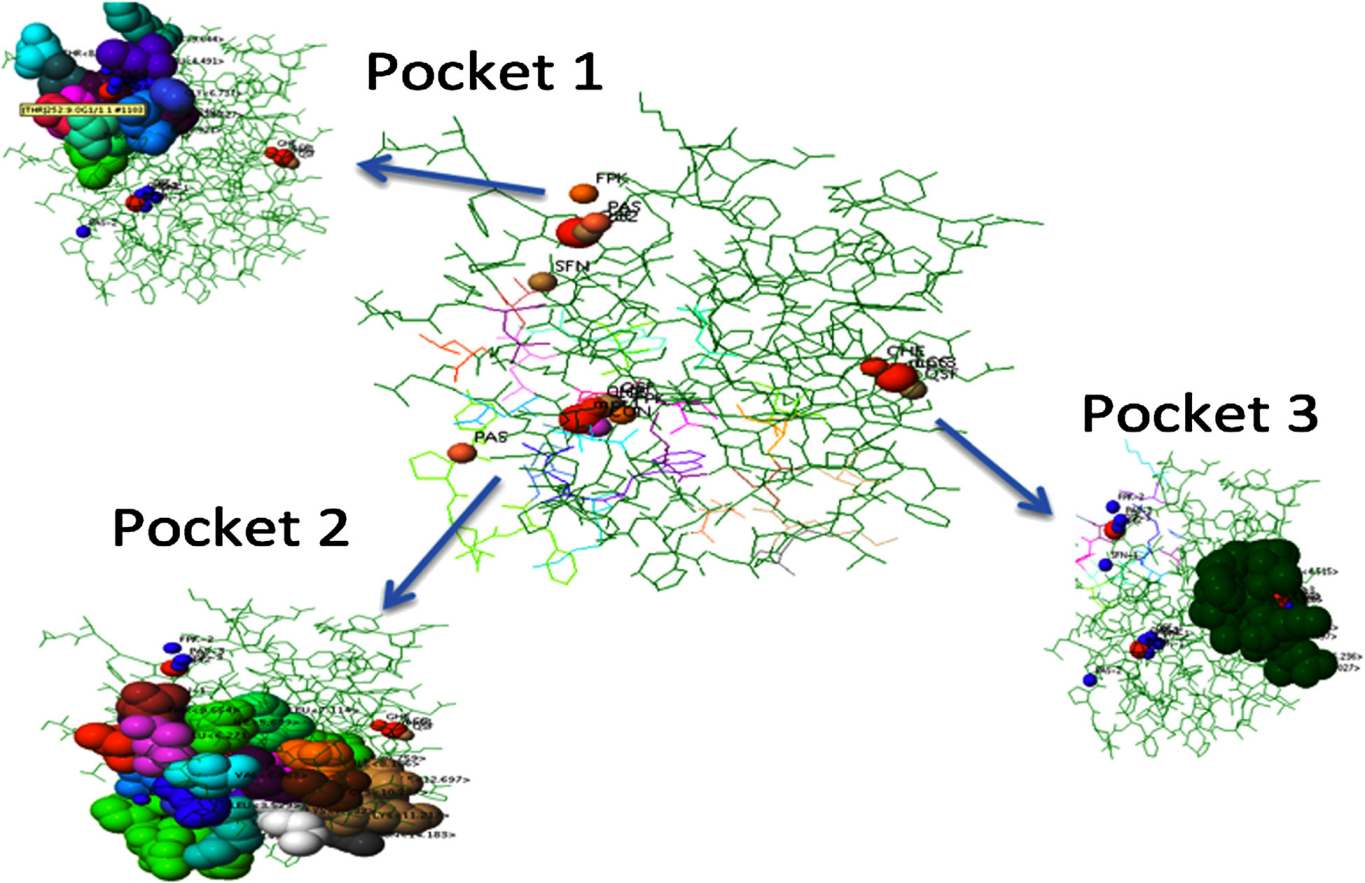
**The predicted potential binding sites in CDT protein of *C. jejuni*.** Pocket color description are indicated as: red - MPK, actinium - PAS, magenta - QSF, potassium - FPK, wheat - SFN, yellow - GHE, blue – CON and raspberry - PCS. The exact residue location information is given in Table 2.

The potential binding sites (PBS) of proteins are those residues or atoms, which bind to ligands directly on protein surface; they are near to the ligand binding sites. After clustering the top three sites from different methods like PAS, QSF, FPK, SFN, GHE, CON, LCS, the MetaPocket 2.0 has predicted seven clusters for the protein structure, but we have presented here three best score pockets sites (Figure 8).

The first MetaPocket site (MPK1) consists of six pocket sites, the first pocket from GHECOM (GHE-1), the first pocket from LigisiteCS (LCS-1), the first pocket from Fpocket (FPK-1), the second pocket from PASS (PAS-2), the first pocket of Q-SiteFinder (QSF-1) and the first pocket from Concavity (CON-1) with total Z-score 11.06 and size of 6. The second MetaPocket site (MPK2) consists of four pockets, from SNF-1, FPK-2, QSF-3 and PAS-3 and the total Z-score is 7.61. The third MetaPocket site (MPK 3) consists of three pocket, from the second pocket of Q-SiteFinder (QSF-2), the third pocket from LigisiteCS (LCS-3), the third pocket from GHECOM (GHE-3) with total Z-score 2.90 and size of 3. Table 2 shows the potential binding sites from a predicted CDT protein of *C. jejuni* in residue. The header binding sites 1, 2 and 3 are designated for MetaPockets 1, 2, 3 respectively. In the case above, potential binding sites of three MetaPockets are given and they are shown in residue format with each line starting with ‘RESI’. The residue described above is constructed in three parts: residue name, chain indicator and residue sequence number.

**Table 2:** Predicted ligand binding site in residues

**Site no**	**Residues**				
**Header binding site 1**	ILE_9^118^	LEU_9^126^	TRP_9^154^	ILE_9^166^	LEU_9^175^
	ILE_9^208^	LEU_9^116^	TRP_9^196^	LEU_9^198^	LEU_9^158^
	ALA_9^164^	MET_9^165^	LYS_9^197^	VAL_9^206^	LEU_9^251^
	ILE_9^217^	LEU_9^156^	THR_9^252^	ASN_9^210^	LYS_9^215^
	ILE_9^234^	CYS_9^216^	ASN_9^213^	LYS_9^209^	ILE_9^182^
	PHE_9^163^	ASP_9^162^	ASN_9^161^	TYR_9^159^	PRO_9^160^
**Header binding site 2**	LEU_9^116^	THR_9^117^	THR_9^252^	THR_9^253^	PRO_9^254^
	PRO_9^255^	ALA_9^125^	LEU_9^142^	ARG_9^152^	LEU_9^119^
	GLY_9^123^	PHE_9^256^	LYS_9^146^	THR_9^257^	
**Header binding site 3**	TRP_9^136^	TRP_9^138^	VAL_9^231^	PHE_9^232^	ASN_9^180^
	GLY_9^181^	LYS_9^233^	GLY_9^179^	ILE_9^182^	ILE_9^137^

## Conclusions

The purpose of the present study was to perform a global screening for new immunogenic HLA class I (HLA-I) restricted cytotoxic T cell (CTL) epitopes of potential utility as a vaccine candidate against campylobacteroisis. The five epitopes of CDTCJ were identified. It is anticipated that, the peptide _170_KTNTCLNAY_178_ can serve as novel potential vaccine candidate against diarrhea. These results have important implications for the rational design of CTL epitope-based CDT campylobacteriosis diagnostics and vaccines applicable to all ethnic groups. The presented research offered a backbone for understanding structural and functional insights of CDT protein. The additional experimental work is required to validate this epitope. The identification of ligand-binding sites is often the starting point for protein function annotation and structure-based drug design. In this study, we identify three predicted potential binding sites in CDT protein of *C. jejuni*. These are active sites on protein surface that performs protein functions.

## Competing interests

The authors declare that they have no competing interest.

## Authors’ contributions

AI designed the study, performed *in silico* works and wrote the manuscript, SG provided a platform and critically reviewed the manuscript. Both authors have read and approved the final manuscript.

## Authors’ information

Dr. Arun Ingale (Associate Professor and Head)

Department of Biotechnology, School of Life Sciences, North Maharashtra University, Jalgaon 425001, India.

Dr. Susumu Goto (Associate Professor)

Bioinformatics Centre, Institute of Chemical Research, Kyoto University, Kyoto, Japan.
